# Association between Paraoxonase 1 (PON1) Polymorphisms and the Risk of Acute Coronary Syndrome in a North African Population

**DOI:** 10.1371/journal.pone.0133719

**Published:** 2015-08-04

**Authors:** Abdelghani Bounafaa, Hicham Berrougui, Noreddine Ghalim, Boubker Nasser, Abdallah Bagri, Abderrahmane Moujahid, Souad Ikhlef, Pamela Camponova, Najoua Yamoul, Olivier Kamtchueng Simo, Abdelkhalid Essamadi, Abdelouahed Khalil

**Affiliations:** 1 Laboratory of Biochemistry & Neuroscience, Applied Biochemistry and Toxicology Team, Faculty of Sciences and Technology, Hassan First University, Settat, Morocco; 2 Department of Medicine, Geriatrics Service, Faculty of Medicine and Biological Sciences, University of Sherbrooke, Sherbrooke, Quebec, Canada; 3 Department of Biology, Polydisciplinary Faculty, Sultan Moulay Sliman University, Beni-Mellal, Morocco; 4 Laboratory of Biochemistry, Pasteur Institute of Morocco, Casablanca, Morocco; 5 Cardiology Service, Ibn Rochd University Hospital Center, Casablanca, Morocco; CSIR-INSTITUTE OF GENOMICS AND INTEGRATIVE BIOLOGY, INDIA

## Abstract

The purpose of the present study was to investigate the distribution of PON1 Q192R and L55M polymorphisms and activities in a North African population and to determine their association with cardiovascular complications. The prevalence of the QQ, QR, RR, LL, LM, and MM genotypes in the study population was 55.4%, 34.09%, 9.83%, 41.97%, 48.20%, and 9.83% respectively. The Q, R, L, and M alleles had a gene frequency of 0.755, 0.245, 0.67, and 0.33, respectively. The PON1 192 RR genotype was significantly more prevalent among ACS patients than among healthy subjects. There was a 4.33-fold increase in the risk of ACS in subjects presenting the PON1 192 RR genotype compared to those with the QQ genotype (OR=4.33; 95% CI=1.27–17.7). There was a significantly different distribution of PON1 L55M in the ACS patient groups (UA, STEMI, NSTEMI). Moreover, individuals presenting the PON1 55MM genotype present a higher risk for ACS than those with LL genotype (OR=3.69; 95% CI=1.61–11.80). Paraoxonase activities were significantly lower in coronary patients than in healthy subjects. The decrease in PON1 activity was inversely correlated with the number of concomitant risk factors for CVD (r=0.57, p<0.0001). The results of the present study suggested that the PON1 R and M alleles may play a role in the pathogenesis of cardiac ischemia in our North African population and that a decrease in PON1 activity may be a valuable marker for monitoring the development of the atherosclerosis process and the associated cardiovascular complications.

## Introduction

Acute coronary syndrome (ACS) is a common complication and a life-threatening form of coronary heart disease (CHD). ACS includes unstable angina (UA), non-ST segment elevation myocardial infarction (NSTEMI), and ST segment elevation myocardial infarction (STEMI). The disruption of atherosclerotic plaque and the resulting intracoronary thrombosis are thought to account for most ACS cases [1,2]. Coronary artery disease (CAD) remains the leading cause of death in most developed countries. According to estimates by the World Health Organization, nearly seven million people worldwide die of CAD each year, with most of these deaths occurring in developing countries [3]. More than 80% of sudden cardiac deaths are caused by atherosclerotic CAD [4].

Atherosclerosis is characterized by the buildup of fatty lesions, inflammation, and scarring of arterial walls, with oxidative stress as a primary contributing factor [5]. The oxidative modifications of low-density lipoproteins (LDL) in the arterial wall may play a major role in the development of atherosclerotic lesions. Oxidative stress is known to increase the formation of oxidized LDL. Early studies on atherosclerosis generally suggested that LDL is the main cause of this pathology.

Human paraoxonase 1 (PON1) is an esterase that catalyzes the hydrolysis of organophosphate paraoxon and that hydrolyzes oxidized lipids, which are involved in the initiation and progression of atherosclerosis [6]. PON1 activity is recognized as an independent risk factor for atherosclerotic vascular diseases [6,7]. PON1 is a high-density-lipoprotein-(HDL)-associated esterase that appears to contribute to the antioxidant and anti-atherosclerotic activities of HDL [8–10]. PON1 is synthesized in the liver and is secreted into the bloodstream where it breaks down both man-made and naturally occurring compounds [5]. PON1 is named for its ability to hydrolyze organophosphates such as paraoxon [11], which are found in insecticides. It also hydrolyzes N-acyl-homoserine, a lactone used by pathogenic bacteria [12], as well as lipid peroxides, inhibiting the formation of foam cells, which are known to contribute to atherosclerosis [13]. Moreover, PON1 exerts its anti-inflammatory properties by hydrolyzing oxidized phospholipids [14], which are modulators of inflammation and which accumulate in atherosclerotic lesions [15,16].

The most studied PON1 gene polymorphisms result from amino acid substitutions at positions 192 (Gln-Arg) and 55 (Leu-Met) in the coding region of the gene. Alleles at the 192 (Q and R alleles) and 55 (L and M alleles) loci of the PON1 codon have been associated with enzyme activity and concentration, respectively [17]. The RR-genotype exhibits a high paraoxonase activity (high activity phenotype), while the QQ-genotype exhibits low paraoxonase activity (low activity phenotype) [18]. However, there is also a marked variation in enzyme activity between individuals of the same genotype [19].

PON1 192 and 55 polymorphisms have been widely investigated, especially for their possible involvement in the onset and severity of CVD [20]. While Mendonca et al. [21] associated these two polymorphisms with the risk of cardiovascular diseases, Wheeler et al. [20] reported no such association. To date, the role of PON1 genetic polymorphisms in CVD remains controversial, and further studies are required to better determine the involvement of the PON1 gene in cardiovascular pathologies.

Data on the distribution frequency of PON1 192 genotypes and the risk of CAD have been reported in several populations around the world [22]. However, fewer studies have investigated this issue in African populations and much less so in North African countries where CVD is increasing in prevalence and becoming a significant cause of premature morbidity and mortality [23]. Moreover, projections by the Global Burden of Disease Project suggest that the burden of CVD faced by African countries will double from 1990 to 2020 [24]. A study by Kallel et al. showed that PON1 Q192R polymorphisms, particularly RR, are an important risk factor for myocardial infarction in the Tunisian population [25]. However, Kallel et al. did not report the level of PON1 activity (PON1 phenotype) in the study population [25]. Interestingly, PON1 activity has been proposed as more predictive of vascular diseases than the PON1 genotype [6,26]. Moreover, several studies have shown that lower PON1 paraoxonase activity is associated with atherosclerotic complications [26–28], indicating that it is important to determine the phenotype, not just the genotype, when studying atherosclerosis.

Clinicians are increasingly turning to genetic approaches for making diagnoses given the inadequacy of international guidelines and cardiac enzyme tests, especially in the case of elderly patients where CVD frequently has an unusual onset and often leads to misdiagnosis. As such, studies to find new molecular markers are clearly warranted. We investigated the phenotypic and genotypic distributions of PON1 and analyzed the relationship between the 55 and 192 polymorphisms and ACS in a North African population.

## Materials and Methods

### Subjects

Three hundred five subjects were enrolled in our study and were distributed into two groups based on health status. The first group consisted of 100 healthy subjects who were recruited from patients visiting the Biomedical Centre of the Casablanca Pasteur Institute in Casablanca, Morocco, for medical check-ups. These subjects (52 men and 48 women, mean age: 54.95 ± 0.56 years) were all healthy non-smokers and were not undergoing any treatments or taking vitamin supplements. The second group consisted of 205 patients with ACS (125 men and 80 women, mean age 57.47 ± 0.67 years), who were enrolled at the Cardiology Service of the University Hospital Center Ibn Rochd in Casablanca, Morocco. They met the diagnostic criteria for ACS, which was characterized using ECGs as STEMI, NSTEMI, or UA. Acute myocardial infarction was confirmed with instrumental examinations, including coronary angiograms and echocardiograms. Patients suffering from hemorrhagic or ischemic stroke, heart failure, arthritis, hypertension, or diabetes were included. Patients with dysthyroidism or renal failure (creatinine clearance <40 mL/min) or undergoing hormonal treatment were excluded. Arterial blood pressures, lipid profiles (LDL, HDL, and total cholesterol), and C-reactive protein (CRP) and glycemia levels were determined. The biochemical and physical characteristics of the healthy subjects and the ACS patients are listed in Table 1. In the absence of an ethics committee, an ad hoc committee formed by the cardiology service of the University Hospital Center Ibn Rochd approved the protocol. All participants provided written informed consent prior to taking part in the study.

**Table 1:** Demographic and clinical data of the healthy subjects and the ACS patients.

Parameter	Healthy subjects	ACS patients	P values
**Mean age (years)**	54.95 ± 0.5551	57.47 ± 0.6699	**<0.05** **
**Number (male/female)**	52/48	125/80	**0.14**
**BMI (kg/m** ^**2**^ **)**	24.5 ± 0.22	27.2±0.261	**<0.001** ***
**Systolic blood pressure**	120.9 ± 0.95	132.8 ± 1.12	**<0.001** ***
**Diastolic blood pressure**	71.00 ± 0.61	77.02 ± 0.77	**<0.001** ***
**Glucose (mmol/L)**	5.14 ± 0.01	8.23 ± 0.049	**<0.001** ***
**TC (mmol/L)**	3.75 ± 0.08	4.68 ± 0.08	**<0.001** ***
**TG (mmol/L)**	1.19 ± 0.03	2.15 ± 0.07	**<0.001** ***
**HDL-C (mmol/L)**	1.25 ± 0.02	0.98 ± 0.02	**<0.001** ***
**LDL-C (mmol/L)**	2.85 ± 0.05	3.72 ± 0.07	**<0.001** ***
**Diabetes**	**0%**	**42%**	
**Family history of ACS**	**0%**	**26%**	
**Smokers**	**0%**	**35%**	
**Statin intake**	**0%**	**5%**	

Values are means ± SEM, unless indicated otherwise. The student’s t-test and χ^2^ test (for sex) were used. Significance was established by comparing the results from the ACS patients with those from the healthy subjects:

** p<0.05,

*** p<0.001.

HDL-C (HDL-cholesterol), LDL-C (LDL-cholesterol), TC (total cholesterol), CRP (C reactive protein), TG (triglycerides)

### Blood sample collection and measurement of lipid profile

Blood samples were collected in dry or EDTA as previously described [29]. The samples were centrifuged at 3000 xg and aliquots of plasma were immediately stored at -80°C until analyzed. Whole blood samples (1 mL) for each subject were kept for polymorphism analyses. Serum total glucose, total cholesterol, HDL-cholesterol, LDL-cholesterol, triglyceride, and C-reactive protein levels were measured using automated enzymatic assays (Kodak, Ektachem USA Systems).

### Oxidative stress markers

Systemic oxidative stress was evaluated by measuring plasma protein carbonyl and malondialdehyde (MDA) levels by HPLC and carbonyl levels by spectrophotometry as previously described [30].

### PON1 activities

PON1 paraoxonase and arylesterase activities were determined by the measurement of the hydrolysis of paraoxon and phenyl acetate as a previously described [29,31]. The ratio of salt-stimulated PON1 paraoxonase and arylesterase activities was used to determine the phenotypic distribution of PON1 [32].

### DNA extraction and PON1 genotype determination

DNA was extracted from granulocytes using the sodium iodide procedure as previously described in Cherki et al. [33]. The PON1 R192Q and L55M genotypes were determined by PCR as previously described [33,34]. For more detail see S1 text.

### Statistical analysis

Comparisons between groups were performed using a t-test for continuous variables or an *χ* ^*2*^ test for categorical variables. Allele frequencies were calculated by allele counting. The concordance of genotype frequencies using the Hardy-Weinberg equilibrium was tested using an χ^2^ goodness-of-fit test. The contribution of the PON1 polymorphisms to ACS was estimated by logistic regression for unmatched data to obtain odds ratios for the PON1 polymorphisms adjusted for the effects of age, sex, and HDL levels. A one-way ANOVA was used for multiple comparisons, and the Pearson correlation was calculated to assess the association between PON1 paraoxonase activity and the number of concomitant CVD risk factors. Values are expressed as percentages or as means ± SEM unless otherwise indicated. All results were interpreted at an alpha level of 0.05.

## Results

### Baseline data

The baseline characteristics of the participants are summarized in Table 1. There was a significant difference between ACS patients and healthy subjects with respect to age, BMI, blood pressure, glycemia, and lipid profile (total cholesterol, triglycerides, and HDL and LDL levels). The ACS patients had lower HDL levels (0.98 ± 0.02 mmol/L, p<0.001) and higher LDL (3.72 ± 0.07 mmol/L, p<0.001) and triglyceride (2.15 ± 0.07 mmol/L, p<0.001) levels than the healthy subjects (Table 1). Approximately 42% of the ACS patients were diabetic, 30% were obese, 35% were cigarette smokers, 35% were hypertensive, and 26% had a family history of ACS.

### PON1 activities in healthy subjects and ACS patients

PON1 paraoxonase activity was determined by measuring the hydrolysis of paraoxon, while PON1 arylesterase activity was measured by the hydrolysis of phenylacetate. The obtained ratio (R) was used to categorize the subjects based on their phenotypes: homozygous QQ (R<3), heterozygous QR, (3<R<7), or homozygous RR (R>7) as described in detail previously [29]. The allelic frequencies of the ACS patients and the healthy subjects based on phenotype are shown in Table 2. The prevalence of the QQ, QR, and RR phenotypes in the entire study population were 53.11%, 34.09%, and 12.79%, respectively. There was a significant difference in the distribution of the RR phenotype between the healthy subjects and the ACS patients (RR = 4% vs.17.07%, respectively, p<0.01).

**Table 2:** PON1 phenotypic distribution, activities, and oxidative stress markers in the healthy subjects and ACS patients.

Participants (n = 305)	Healthy subjects (n = 100)	ACS patients (n = 205)
**QQ 53.11% (n = 162)**	57% (n = 57)	51.22% (n = 105)
**QR 34.09% (n = 104)**	39% (n = 39)	31.71% (n = 65)
**RR 12.79% (n = 39)**	4% (n = 4)	17.07%** (n = 35)
**MDA (μM)**	2.35 ± 0.17	7.12 ± 0.30****
**Protein carbonyl levels (nmol/mg)**	3.07 ±0.17	9.29 ± 0.26****
**CRP (mg/L)**	6.78 ± 0.34	10.11 ± 0.76***
**Paraoxonase activity (U/ml)**	366.3 ± 16.12	210.1 ± 6.37***
**Arylesterase activity (U/ml)**	131.7 ± 4.73	78.67 ± 3.22***

Values are means ± SEM, unless indicated otherwise. The Student’s *t-test* and χ2 test (for PON1 phenotypic distribution) were used. Significance was established by comparing the results from the ACS patients with those from the healthy subjects:

**p<0.01,

***p<0.001,

****p<0.0001.

**Fig 1:**
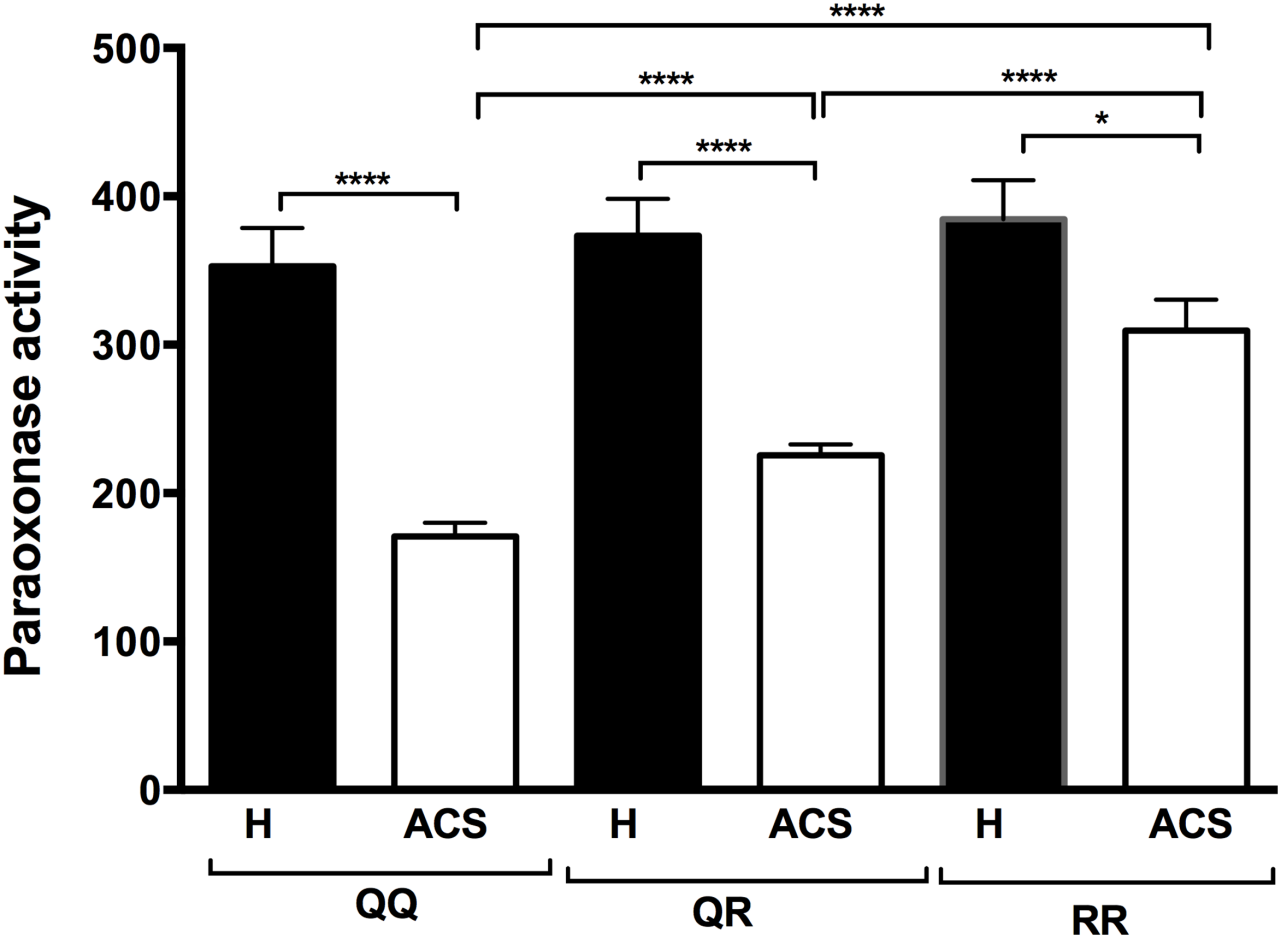
PON1 paraoxonase activities in the healthy subjects and the ACS patients based on their PON1 192 genotype. The PON1 Q192R genotype was determined by RT-PCR. PON1 paraoxonase activity was determined by measuring paraoxon absorbance at 412 nm. Results are expressed as means ± SEM. *p<0.03 and ****p<0.0001 for comparison between ACS patients and healthy subjects with the same PON1 Q192R polymorphism or comparison between PON1 Q192R polymorphisms within the ACS group.

Our results showed that paraoxonase and arylesterase activities were significantly lower in the ACS patients than in the healthy subjects (Table 2). The lower PON1 paraoxonase activity in the ACS patients was independent of the PON1 192 genetic polymorphism. Indeed, paraoxonase activity was significantly lower in the ACS patients for all three PON1 192 genotypes compared to the healthy subjects (Fig 1). PON1 paraoxonase activity was also significantly different between the three PON1 Q192R polymorphisms in the ACS patients (Fig 1), while no significant differences were observed between these polymorphisms in the healthy control group.

**Fig 2:**
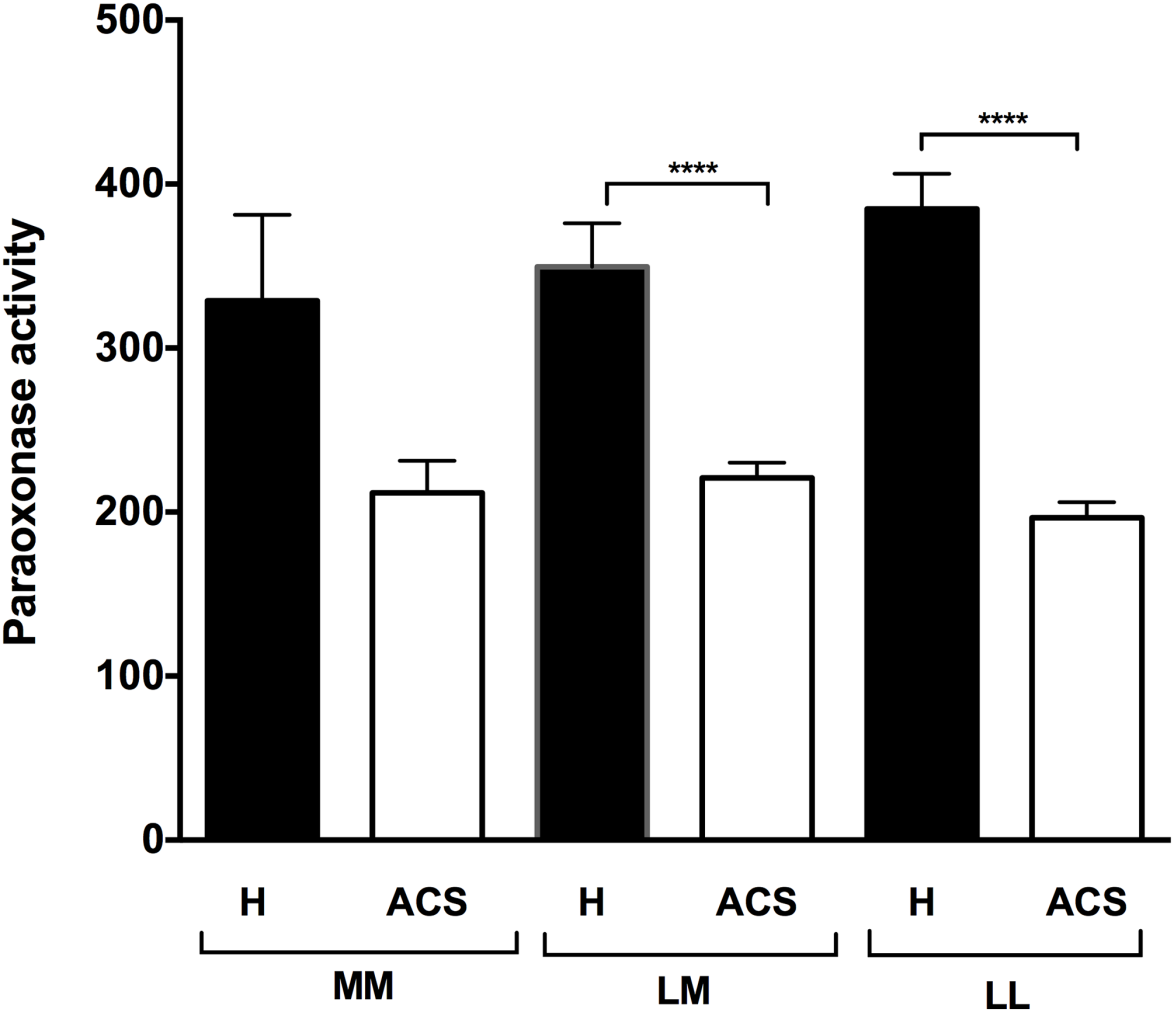
PON1 paraoxonase activities in the healthy subjects and the ACS patients based on their PON1 55 genotype. The PON1 L55M genotype was determined by RT-PCR. PON1 paraoxonase activity was determined by measuring paraoxon absorbance at 412 nm. Results are expressed as means ± SEM. ****p<0.0001 for comparison between ACS patients and healthy subjects with the same PON1 L55M polymorphism.

**Fig 3:**
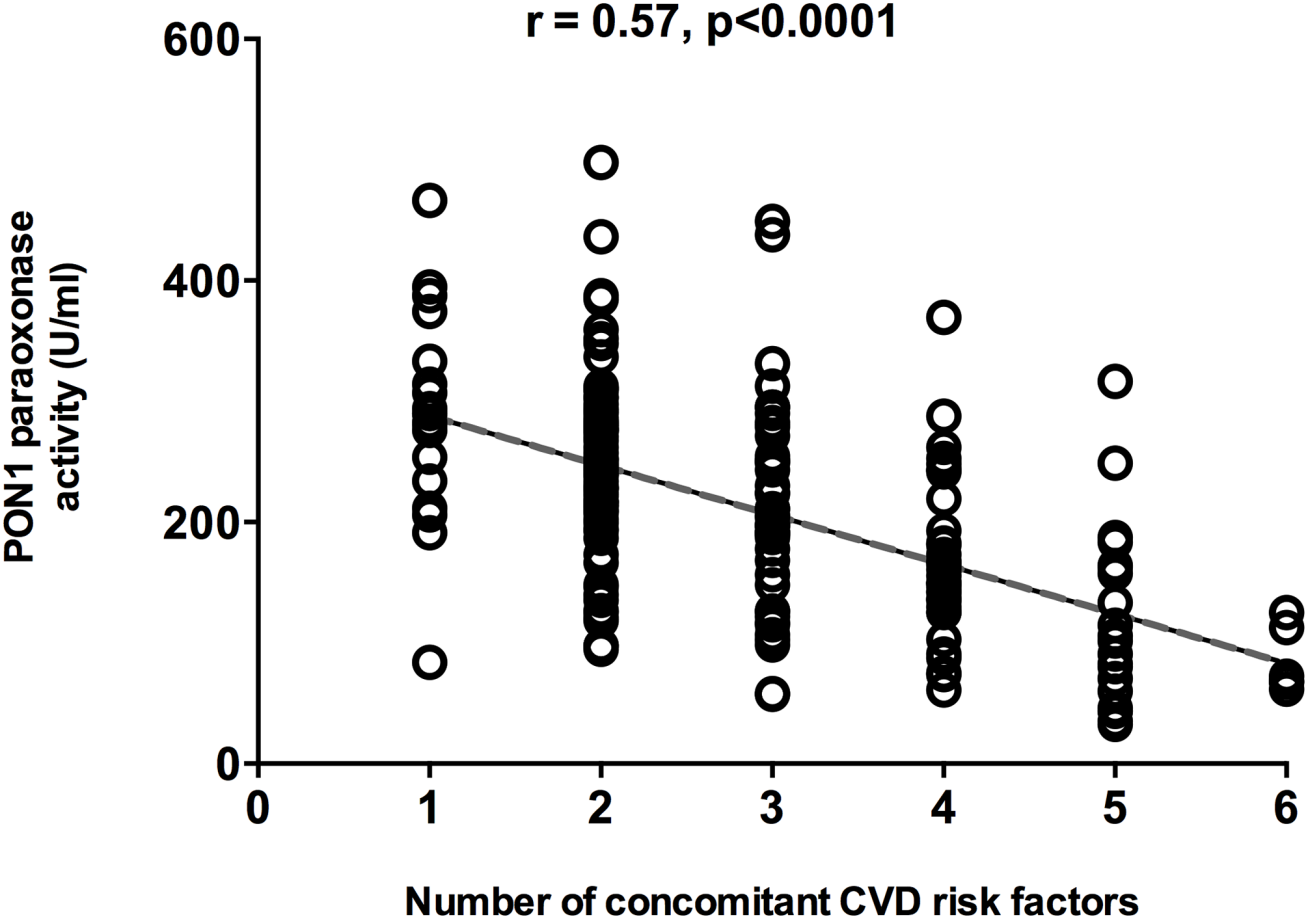
Correlation between PON1 paraoxonase activity and the number of concomitant risk factors for CVD. CVD risk factors: diabetes, obesity, high arterial blood pressure, alcohol, smoking, and family history. A Pearson correlation analysis was performed to assess the association between CVD risk factors and PON1 paraoxonase activity. H: Healthy, ACS: Acute Coronary Syndrome.

In terms of the PON1 55 genetic polymorphisms, PON1 paraoxonase activity was also significantly lower in the ACS patients than in the healthy subjects for both the LM and the LL polymorphisms (p<0.001) (Fig 2). Our results also showed that there is significantly lower paraoxonase activity in ACS patients presenting each of the CVD risk factors separately (Table 3). Interestingly, the lower PON1 paraoxonase activity in the ACS patients was inversely correlated with the number of concomitant CVD risk factors (diabetes, hypertension, obesity, smoking, excessive alcohol consumption, and family history of CVD (r = 0.57, p<0.0001) (Fig 3).

**Table 3:** PON1 paraoxonase activity as a function of the health status of the participants.

Health status (n = number of subjects)	PON1 activity (U/mL)
Healthy (n = 100)	366.3 ± 16.12
Diabetes (n = 87)	189.93 ± 9.14****
Hypertension (n = 71)	197.52 ± 9.77****
Obesity (n = 57)	215.37 ± 11.06****
Smoking (n = 72)	200.14 ± 10.16****
Alcohol consumption (n = 25)	173.3 ± 16.14****
Family history of CVD (n = 59)	217.97 ± 12.04****

Values are means ± SEM. Significance was established by comparing the health status of the ACS group to that of the healthy control group.

**** p<0.001 for comparisons with the healthy control group using the student’s *t-test*.

**Fig 4:**
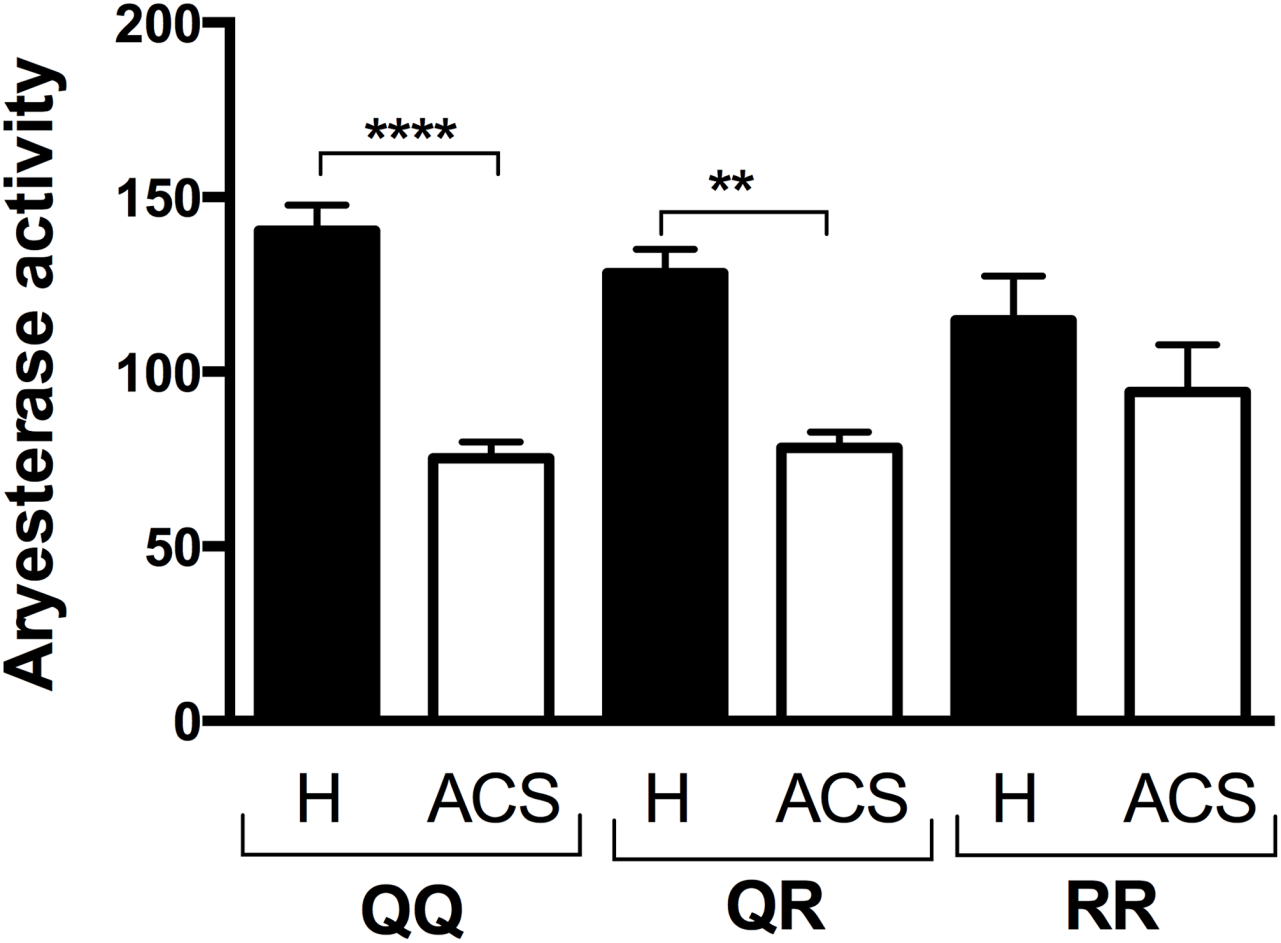
PON1 arylesterase activity in the healthy subjects and the ACS patients and based on their PON1 192 genotype. The PON1 Q192R genotype was determined by RT-PCR. PON1 arylesterase activity was measured by the increase in absorbance at 270 using phenylacetate as a substrate. Results are expressed as means ± SEM. ** p<0.001 and ****p<0.0001 for the ACS patients compared to the healthy subjects with the same PON1 Q192R polymorphism.

**Fig 5:**
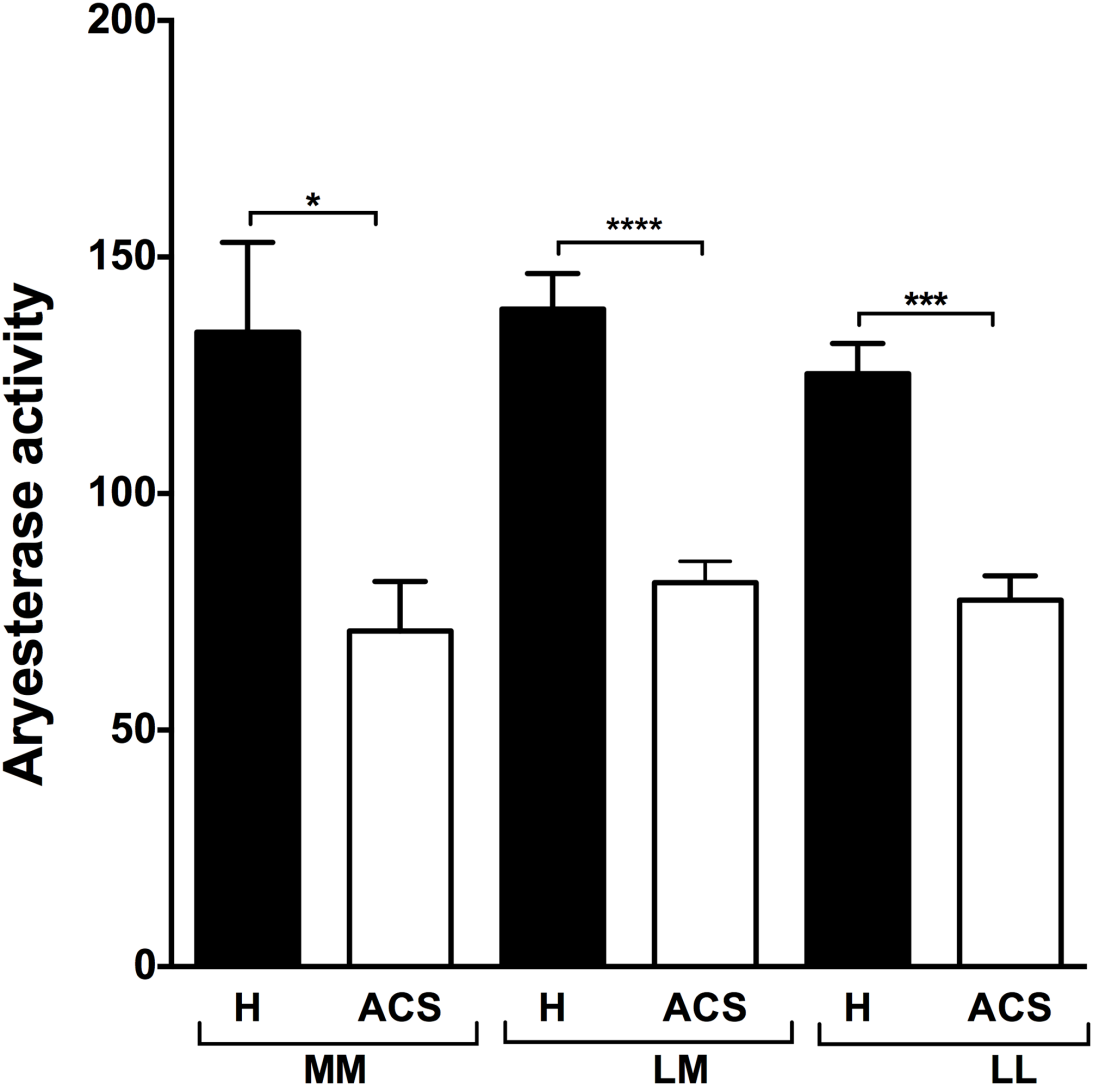
PON1 arylesterase activity in the healthy subjects and the ACS patients and based on their PON1 55 genotype. The PON1 L55M genotype was determined by RT-PCR. PON1 arylesterase activity was measured by the increase in absorbance at 270 using phenylacetate as a substrate. Results are expressed as means ± SEM. *p<0.03, ** p<0.001 and ****p<0.0001 for the ACS patients compared to the healthy subjects with the same PON1 L55M polymorphism.

PON1 arylesterase activity was also significantly lower in the ACS patients than in the healthy subjects (Table 2) for both the 192 and the 55 polymorphisms (Figs 4 and 5). However, no significant difference was observed between the healthy subjects and the ACS patients for the RR polymorphism (Fig 4).

### Genotype distributions and allele frequencies of the Q192R and L55M polymorphisms

The PON1 Q192R and L55M polymorphisms were genotyped by RT-PCR. The genotypic distributions of PON1 Q192R in the healthy subjects and the ACS patients are presented in Table 4. The prevalence of the QQ, QR, and RR genotypes in the entire study population was 55.4%, 34.09% and 9.83%, respectively, while the gene frequencies of the Q and R alleles were 0.755 and 0.245, respectively, which is in excellent agreement with the Hardy-Weinberg equilibrium. Importantly, the distribution of the PON1 Q192R genotype determined by RT-PCR was comparable to the phenotypic distribution of PON1 obtained using the two-substrate method.

**Table 4:** PON1 genotypic distribution and odds ratios of the genotype and alleles of the Q192R polymorphism in the healthy subjects and the ACS patients.

Genotype	Healthy subjects	ACS patients	Odds ratio	P value
			(95% CI)	
QQ (n = 169) 55.4%	57% (n = 56)	55.12% (n = 113)	Reference	
QR (n = 106) 34.09%	39% (n = 39)	32.68% (n = 67)	0.867 (0.522–1.439)	n.s.*
			***1*.*05 (0*.*597–1842)***	***n*.*s***.
RR (n = 30) 9.83%	5% (n = 5)	12.19% (n = 25)	3.153 (1.04–9.49)	<0.041
			***4*.*33 (1*.*27–17*.*7)***	***0*.*019***
Allele				
Q	75.50% (n = 151)	71.46% (n = 293)	Reference	
R	24.50% (n = 49)	28.53% (n = 117)	1.3 (0.879–1.922)	<0.19

*n.s., not significant

Contribution of the PON1 Q192R polymorphism to ACS was estimated by logistic regression for unmatched data to obtain odds ratios for the PON1 Q192R polymorphisms. ***Italics bold indicate the odds ratios adjusted for age*, *sex*, *BMI and HDL cholesterol*.**

The distribution of the QQ and RR genotypes was significantly different between the healthy subjects and the ACS patients (p = 0.041). The determination of the association of the PON1 Q192R genotype with the risk of ACS showed that there was a 3.15-fold increase in ACS risk in subjects presenting the RR genotype compared to the QQ genotype (OR = 3.153; 95% CI = 1.04–9.49) and still significant although when adjusted for age, sex and HDL cholesterol adjusted for age, sex, BMI and HDL cholesterol (OR = 4.33; 95% CI = 1.27–17.7) (Table 4).

The distributions of the PON1 L55M genotype in the healthy subjects and the ACS patients are presented in Table 5. The prevalence of the LL, LM, and MM genotypes in the entire study population was 41.9%, 48.2%, and 9.83%, respectively. The L and M alleles had a gene frequency of 0.67 and 0.33, respectively, which is in excellent agreement with the Hardy-Weinberg equilibrium. Subjects presenting MM genotype present a higher risk for ACS when compared to LL genotype (OR = 2.74, 95% CI = 1.04–7.16) and still significant although when adjusted for age, sex and HDL cholesterol (OR = 3.693, 95% CI = 1.61–11.18).

**Table 5:** Genotype and allele frequencies of the L55M polymorphism.

PON1 genotype	Healthy subjects	ACS patients	Odds ratio	p-value
	(n = 100)	(n = 205)	(95% CI)	
**PON1 55**				
**LL (41.97%)** *	51% (n = 52)	37.56% (n = 76)	reference	
**LM (48.20%)** *	43% (n = 42)	51.22% (n = 105)	1.71 (1.04–2.83)	0.036
			***1*.*49 (0*.*82–2*.*71)***	***0*.*19***
**MM (9.83%)** *	6% (n = 6)	11.71% (n = 24)	2.74 (1.04–7.16)	0.04
			***3*.*693 (1*.*61–11*.*8)***	***0*.*027***
**Allele L (Leu)**	73% (n = 146)	62.68% (n = 257)	reference	
**Allele M (Met)**	27% (n = 54)	37.32% (n = 153)	1.61 (1.11–2.33)	0.012

Contribution of the PON1 L192M polymorphism to ACS was estimated by logistic regression for unmatched data to obtain odds ratios for the PON1 L192M polymorphisms. ***Italics bold indicate the odds ratios adjusted for age*, *sex*, *BMI and HDL cholesterol*.**

*(%) of the entire population (healthy subjects and ACS patients)

**Fig 6:**
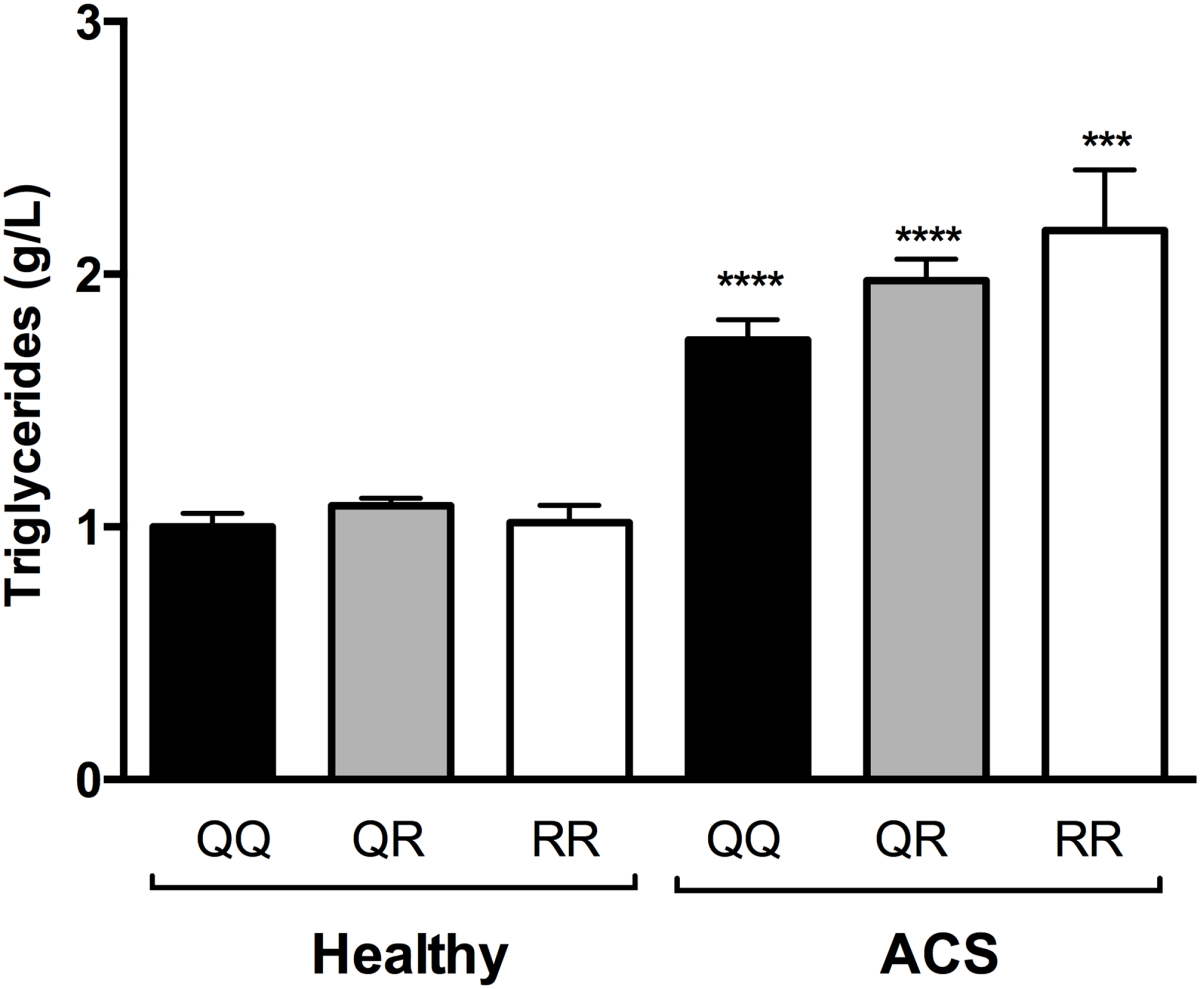
Comparison of the triglyceride levels of healthy subjects and ACS patients as a function of PON1 Q192R polymorphism. ***p<0.0001 and **** p<0.001 compared with the healthy subjects with the same PON1 Q192R polymorphism.

**Fig 7:**
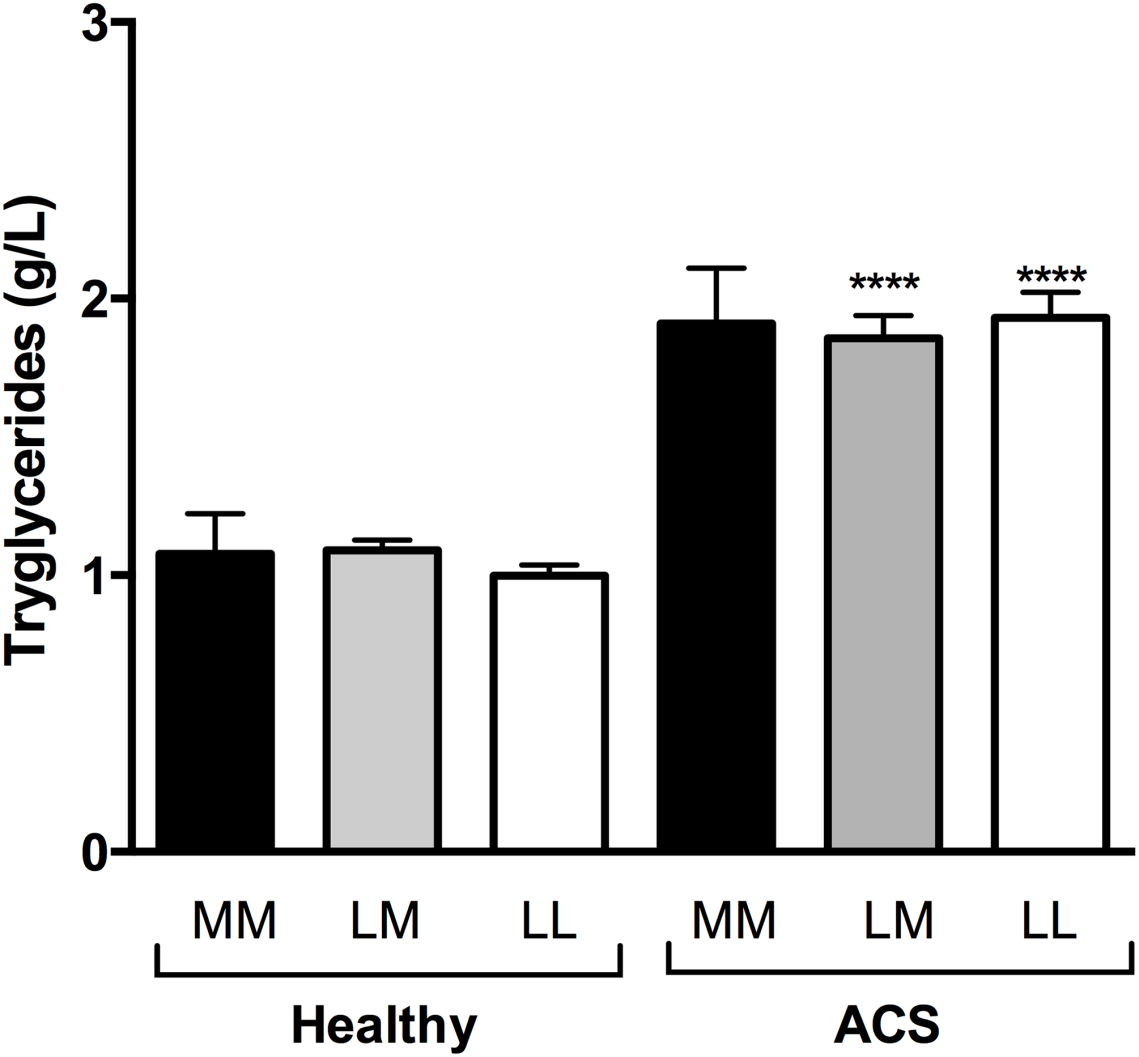
Comparison of the triglyceride levels of healthy subjects and ACS patients as a function of PON1 L55M polymorphism. **** p<0.001 compared with the healthy subjects with the same PON1 L55M polymorphism.

The frequency distributions of the PON1 55 and PON1 192 carriers based on the diagnosis of UA, STEMI, or NSTEMI are reported in Table 6. A comparison of carrier frequency distributions showed that there was a significant different among the three groups of ACS patients (χ^2^ = 17.2, d.f. = 2, *p* = 0.0002 for PON1 55). The assessment of the mean plasma levels of malondialdehyde, protein carbonyl, triglycerides, total cholesterol, and CRP showed that ACS patients carrying the PON1 55 LL genotype had significantly higher CRP plasma levels than the PON1 55ML/MM carriers (Table 7). Interestingly, the same tendency was observed for malondialdehyde, protein carbonyl, and total cholesterol levels in the LM+MM carriers, although it was not significant (Table 7). Moreover, carriers of the PON1 192 QQ genotype in ACS group displayed significantly lower malondialdehyde and triglycerides plasma levels than carriers of the QR-RR genotype (Table 7). Interestingly, the ACS patients had higher triglyceride levels than the healthy subjects for both the PON1 Q192R and the L55M polymorphisms (Figs 6 and 7).

**Table 6:** PON1 55 and PON1 192 carrier frequencies of the 205 ACS patients.

ACS diagnosis	P192: (QQ)	(QR+RR)	L55: (LL)	(LM+MM)
**UA (unstable angina)**	14 (63.6%)	8 (36.4%)	8 (36.6%)	14 (63.6%)
**STEMI (ST-elevation myocardial infarction)**	85 (53.1%)	75 (46.9%)	61 (38.1%)	99 (61.9%)
**NSTEMI (No ST-elevation myocardial infarction)**	14 (60.8%)	9 (39.1%)	8 (34.7%)	15 (65.3%)
**Total**	113 (55.1%)	92 (44.9%)	77 (37.6%)	128 (62.4%)

χ^2^ = 2.83, d.f. = 2, *p* = 0.09 for comparisons between the ACS diagnosis and PON192 carriers, and χ^2^ = 17.2, d.f. = 2, *p* = 0.0002 for comparisons between the ACS diagnosis and PON55 carriers

**Table 7:** Levels of biochemical parameters of PON1 55 and PON1 192 carriers in the ACS patient group.

Biochemical parameters	LL	ML+MM	*P*	QQ	QR+RR	*P*
**Malondialdehyde (μM)**	7.3±0.48	6.87±0.38	0.49	6.56±0.5	7.54±0.37	**0.01**
**Protein carbonyl (nmol/mg)**	9.49±0.36	8.92±0.35	0.29	8.7±0.4	9.72±0.34	0.055
**Triglycerides (g/L)**	1.86±0.07	1.93±0.09	0.59	1.74±0.07	2.01±0.08	**0.02**
**Total cholesterol (g/L)**	4.73±0.01	4.63±0.12	0.5	4.61±0.12	4.76±0.10	0.36
**C-reactive protein (mg/L)**	11.42±0.92	8.88±0.59	**0.016**	10.09±0.76	9.6±0.7	0.63

The unpaired student’s t-test was used.

## Discussion

Atherosclerosis and related diseases such as stroke and CVD are major causes of mortality and morbidity in developed countries. While diabetes, smoking, and obesity are established risk factors for atherosclerosis complications [35], several studies have suggested that PON1 may play an atheroprotective role [9,10]. However, simultaneous associations between PON1 polymorphisms and enzyme activity with CVD risk have been reported in a limited number of population studies [9,36–41]. The purpose of the present study was to investigate PON1 phenotype and genotype distributions in a Moroccan population and to determine whether the Q192R and L55M polymorphisms in the PON1 coding region can predict CVD complications in this population.

The PON1 Q192R phenotype had a trimodal distribution, with low, intermediate, and high activity phenotypes (QQ 53.11%, QR 34.09%, and RR 12.79%). This phenotype distribution, which was comparable to those reported in the literature [29,42,43], was determined using a dual-substrate method and was confirmed by an RT-PCR analysis of the PON1 genotype. The results indicated that the enzymatic method may be a simple and rapid technique for determining the PON1 phenotype [37].

The results of the present study showed that the frequency of the PON1 192Q allele is higher than that of PON1 192R (0.755 and 0.245, respectively). The frequency of the alleles was similar to that in some Asian, Indian, Saudi Arabian, and European Caucasian populations [44,45], while the frequency of the PON1 192Q allele was significantly higher than in some African black populations [46].

Some case-controlled studies have shown that the PON1 R allele is very common in CHD patients [21,45,47], indicating that the PON1192 polymorphism may be a risk factor for atherosclerosis [48,49]. Several other studies have shown that there is a significant association between the R allele of the PON1 polymorphism and the development of CAD [25,50]. Our results also showed that individuals carrying the RR genotype have a higher risk for CVD. Previous studies, including ours, have suggested that the PON1 RR polymorphism provides the lowest protection against LDL and HDL oxidation compared to other PON1 polymorphisms [33,51]. Moreover, PON1 confers antioxidant activity on HDL, which decreases in the following order of PON1 genotypes (QQ > QR > RR), with almost no antioxidant activity associated with the RR genotype [51]. While the susceptibility of HDL to lipid peroxidation and the antioxidant effect of HDL were not measured in our population, the increased risk of CVD in subjects carrying the RR genotype could be attributed to an acceleration of the atherosclerotic process in these subjects, that is, an increased susceptibility of LDL to oxidation, a reduction in the antioxidant effect of HDL, and an alteration of their functionality [30]. Our results also showed that the RR and QR patients had significantly higher plasma MDA levels than the QQ patients (p<0.05), which lends credence to the hypothesis that RR patients have a lower HDL antioxidant effect, which in turn could explain why the R allele may be a risk factor for CHD [52,53]. Conversely, several other studies that investigated the association between PON1 Q192R polymorphisms and CAD risk have given inconsistent results [54–57]. A weak association between the R allele and increased CVD risk has been confirmed in most case-control studies and has been supported by meta-analyses [20,58]. Other studies have found no such association [59–61] or found a higher frequency of the R allele among obese (BMI>30 kg/m^2^) compared to normal-weight pre-menopausal women [62]. While increased blood pressure has been associated with the R allele in women over 60 years of age [58], other studies have shown that the common PON1 R192 allele may be a genetic risk factor for atherogenesis by inducing chronic low-grade inflammation [63]. These discrepancies in the association of the PON1 Q192R genotype with CAD risk may be due in part to differences in ethnicity, sample size, gene-gene and gene-environmental interactions, and the genotyping methods used [45].

Interestingly, our data showed that ACS patients carrying the QR and RR genotypes had significantly high triglyceride levels when compared to ACS patients carrying QQ genotype. These results are in agreement with previous studies showing an association between the RR genotype of PON1 and serum triglyceride levels [64,65]. Saha et al. demonstrated that PON1 polymorphisms affect the lipid profile and show that the average allelic effect of PON1 was about 22% for serum triglycerides [64]. Moreover, Ombres et al. have shown that the 192QQ genotype is associated to less atherogenic lipid profile [54]. The mechanism by which PON1 polymorphisms influence the serum triglyceride level is not clearly established. Ombres et al. hypothesized that low serum PON1 activity alleles are associated with a decreased transfer of lipids between HDL and VLDL or LDL [54]. However, further studies are needed to clarify this question.

Our results also showed that individual carrying the MM genotype have a higher risk for ACS. Moreover, there was a significantly different genotypic distribution of PON1 L55M carriers in the three groups of ACS patients (UA, STEMI, and NSTEMI) in our population. The possible role played by the PON1 L55M polymorphism in the onset of CHD has been extensively investigated. Taskiran et al. suggested that the PON1 L55M polymorphism has a significant relationship with CAD with a higher frequency of PON1 55M allele in CAD patients [61]. Kallel et al. have not found a significant effect of PON1 L55M polymorphism on the risk of ACS in Tunisian population [25], which in agreement with the results of a meta-analysis study [20,61]. Interestingly, Barbieri et al. [66] reported that the LL genotype is associated with severe insulin resistance (IR), suggesting that IR might be the missing link between the PON1 L55M polymorphism and increased cardiovascular risk. Martinelli et al. [67] showed that the L55 allele is associated with CAD in a group with metabolic syndrome, while Malin et al. [68] reported that LL homozygous men have more atherosclerotic plaques and complicated lesions in the common iliac arteries than M allele carriers.

Our measurements of PON1 activities showed that PON1 paraoxonase activity is significantly lower in ACS patients than in healthy subjects. Interestingly, the lower PON1 paraoxonase activity was associated with all three PON1 genotypes (QQ, QR, and RR). Importantly, PON1 arylesterase activity was also lower in the ACS patients than in the healthy subjects (78.67 ± 3.22 U/mL *vs*. 131.7 ± 4.73 U/mL, p<0.001). Our results showed that the lower PON1 activity was independent of the PON1 192 and 55 polymorphisms (Figs 2 and 3), which suggests that the lower PON1 activity could not been explained by a difference in the genotypic distribution between healthy and ACS patients. Several studies have shown that PON1 paraoxonase activity decreases with aging [69] and diabetes [70] and is lower in smokers [71]. Moreover, a recent study showed that there is significantly lower PON1 paraoxonase activity in ACS patients [72]. This raises the question as to whether the lower PON1 activity is the cause or the consequence of the cardiovascular complications. While our results cannot clearly answer this question, we did show that the lower PON1 activity depended on the number of concomitant CVD risk factors (diabetes, obesity, high arterial blood pressure, alcohol, smoking, and ACS family history) (Fig 3), that is, the greater the number of concomitant CVD risk factors, the lower the PON1 paraoxonase activity. This suggested that the decrease in PON1 activity may contribute, over the long-term, to the development of ACS, which is in agreement with other studies suggesting that PON1 activity can be used as a CVD prediction marker [26,73].

In summary, our results showed that the PON1 Q192R genotype and allele frequencies in the North African population are similar to those observed in other populations, except for African black populations. The RR genotype was more frequent in the ACS patients than in the healthy subjects. PON1 paraoxonase and arylesterase activities were also significantly lower in the ACS patients than in the healthy subjects, and the lower PON1 paraoxonase activity was significantly correlated with the number of concomitant CVD risk factors. The results of the present study suggested that the PON1 R and M alleles may play a role in the pathogenesis of cardiac ischemia in our North African population. However, such an association between PON1 genotypes and the risk of CVD remains controversial [74]. On the other hand, there is currently a broad consensus on the link between the lower PON1 paraoxonase activity and the risk of developing CVD, which strengthens the suggestion that lower PON1 activity could be used as a marker of the development of the atherosclerosis process and associated cardiovascular complications.

The present study had several limitations that must be taken into account. First, the study cohorts were relatively small, and the ACS group was heterogeneous and included patients suffering from hemorrhagic or ischemic stroke, heart failure, arthritis, hypertension, or diabetes. Second, the healthy subjects were younger than the ACS patients and were not sex- and age-matched with respect to the ACS patients. Nonetheless, the age difference between the healthy subjects and the ACS patients, while significant, did not exceed 2.5 years. Third, the PON1 protein concentration was not measured, which would have contributed to better explain the reduction of PON1 enzymatic activity in the ACS patient group. Fourth, insulin and HOMA-IR values were not measured, which would have allowed us to better define the patients. However, information on the presence of diabetes, smoking, family history of ACS, and statin intake were reported for all subjects.

## Supporting Information

S1 Text Methodological details. (DOCX)
