# Supplementary material for: Association between Paraoxonase 1 (PON1) Polymorphisms and the Risk of Acute Coronary Syndrome in a North African Population
Source: PLoS One. 2015 Aug 4;10(8):e0133719. doi: 10.1371/journal.pone.0133719 (PMC4524730; doi:10.1371/journal.pone.0133719)
Supplement: S1 Text — (DOCX) [file pone.0133719.s001.docx]

**Association between paraoxonase 1 (PON1) polymorphisms and the risk of acute coronary syndrome in a North African population**

Abdelghani Bounafaa^1,2^, Noreddine Ghalim^3^, Hicham Berrougui^2,4^, Boubker Nasser^1^, Abdallah Bagri^1^, Abderrahmane Moujahid^1^, Souad Ikhlef^2^, Pamela Camponova^2^, Najoua Yamoul^5^, Olivier Kamtchueng Simo^2^, Abdelkhalid Essamadi^1^, Abdelouahed Khalil^2*^,

^1^Laboratory of Biochemistry & Neuroscience, Applied Biochemistry and Toxicology Team, Faculty of Sciences and Technology, Hassan First University, Settat, Morocco

^2^Department of Medicine, Geriatrics Service, Faculty of Medicine and Biological Sciences, University of Sherbrooke, Sherbrooke, QC, Canada

^3^Laboratory of Biochemistry, Pasteur Institute of Morocco, Casablanca, Morocco

^4^Department of Biology, Polydisciplinary Faculty, Sultan Moulay Sliman University, Beni-Mellal, Morocco

^5^Cardiology Service, Ibn Rochd University Hospital Center, Casablanca, Morocco

**S1 text:** **Methodological details**

**PON1 activities**

PON1 paraoxonase activity was determined by measuring the absorbance at 412 nm (formation of 4-nitrophenol) using paraoxon (O,O-diethyl-O-p-nitrophenylphosphate; Sigma) as a substrate [1]. Enzymatic activity was calculated using the 17,100 M^-1^cm^-1^ molar extinction coefficient. One unit of paraoxonase activity was defined as 1 nmoL of 4-nitrophenol formed per minute.

PON1 arylesterase activity was determined by measuring the increase in absorbance at 270 using phenylacetate as a substrate [2] Gbandja. Enzymatic activity was calculated using the 1310 M^-1^cm^-1^ molar extinction coefficient. One unit of arylesterase activity was defined as 1 μmoL of phenylacetate hydrolyzed per minute.

**Paraoxonase phenotype distribution**

The phenotypic distribution of PON1 was determined using the double-substrate method, which calculates the ratio of salt-stimulated PON1 paraoxonase and arylesterase activities using paraoxon and phenylacetate as substrates, respectively [3]. The genetic polymorphism at codon 192 QR is responsible for the presence of two isotypes: low activity and high activity. The ratio of paraoxon hydrolysis in the presence of 1 M NaCl (salt-stimulated PON1 activity) to the hydrolysis of phenylacetate was used to assign individuals to one of three possible phenotypes [4]: QQ (homozygous low activity), QR (heterozygous intermediate activity), and RR (homozygous high activity). The cut-off values for assigning a participant to a phenotype were <3.0 for QQ, 3.0 to 7.0 for QR, and >7.0 for RR.

**DNA extraction**

DNA was extracted from granulocytes using the sodium iodide procedure as previously described in Cherki et al. [5]. Briefly, 2 mL of lysis buffer (15 mM Tris, 7.5 mM EDTA, 0.225 mM desferrioxamine, pH 8) was added to the granulocyte suspension. After mixing, 35 μL of 10% SDS was added. The mixture was vigorously mixed to lyse the nuclear membrane. Ten μL of RNase (100 mg/mL) in RNase buffer (10 mM Tris, 1 mM EDTA, 2.5 mM desferrioxamine, pH 7.4) was added, and the samples were incubated for 15 min at 50°C. Thirty μL of Qiagen protease (20 mg/ml in deionized water) was then added, and the samples were incubated at 37°C for an additional 1 h. Subsequently, 0.6 mL of NaI solution (7.6 M NaI, 40 mM Tris, 20 mM EDTA, 0.3 mM desferrioxamine, pH 8.0) and 1 mL of 2-propanol were added. The DNA was precipitated by gently inverting the tubes several times. The DNA was recovered by centrifuging at 5000xg for 15 min at 4°C, washing with 1 mL of 40% 2-propanol, and centrifuging at 5000xg for 5 min. The DNA pellet was washed with 1 mL of 70% EtOH, recovered by centrifugation, and dissolved in 0.1 mM desferrioxamine.

**PON1 genotype determination**

The PON1 R192Q genotypes were determined by PCR as previously described [5,6]. The sense 5’TATTGTTGCTGTGGGACCTGAG3’ and anti-sense 5’CACG CTAAACCCAAATACATCTC3’ primers, which encompass the 192 polymorphic region of the human PON1 gene, were used. The PCR mixture contained 200 ng of DNA template, 0.5 μM sense primer, 0.5 μM anti-sense primer, 200 μM dNTPs, and 1 U of Taq DNA polymerase (New England Biolabs, Canada). The DNA was denatured for 5 min at 95°C. The PCR protocol was as follows: 46 denaturing cycles (1 min at 94°C), a 30-s annealing step at 61°C, and a 1-min extension step at 72 °C. The 99-bp PCR product was digested with 5 U of AlwI restriction endonuclease (New England Biolabs) for 4 h at 37°C. The digestion products were separated on 2% agarose gels and were visualized using SYBR Green (Sigma). The R genotype (arginine) contains a single AlwI restriction site, which results in 66- and 33-bp products. The Q genotype (glutamine) does not contain an AlwI restriction site, which allows the PON1 192 genotype to be determined [5].

For the PON1 55 polymorphism, the PCR mixture and cycling were the same as described above except that 40 cycles were performed. The PCR product (170 bp) was digested with Nla 111 (New England Biolabs) in the presence of BSA (4 h, 37°C), and the digestion products were separated and identified as described above. Allele L (leucine) did not contain the Nla 111 site whereas allele M (methionine) contained the Nla 111 site and gave rise to 126- and 44-bp products.

**References:**

1. Gbandjaba NY, Ghalim N, Hassar M, Berrougui H, Labrazi H, et al. (2012) Paraoxonase activity in healthy, diabetic, and hemodialysis patients. Clinical biochemistry 45: 470-474.

2. Paragh G, Asztalos L, Seres I, Balogh Z, Locsey L, et al. (1999) Serum paraoxonase activity changes in uremic and kidney-transplanted patients. Nephron 83: 126-131.

3. Eckerson HW, Wyte CM, La Du BN (1983) The human serum paraoxonase/arylesterase polymorphism. Am J Hum Genet 35: 1126-1138.

4. Seres I, Paragh G, Deschene E, Fulop T, Jr., Khalil A (2004) Study of factors influencing the decreased HDL associated PON1 activity with aging. Exp Gerontol 39: 59-66.

5. Cherki M, Berrougui H, Isabelle M, Cloutier M, Koumbadinga GA, et al. (2007) Effect of PON1 polymorphism on HDL antioxidant potential is blunted with aging. Experimental gerontology 42: 815-824.

6. Loued S, Berrougui H, Componova P, Ikhlef S, Helal O, et al. (2013) Extra-virgin olive oil consumption reduces the age-related decrease in HDL and paraoxonase 1 anti-inflammatory activities. Br J Nutr 110: 1272-1284.
